# Assessing the impacts of in-feed probiotic on the growth performance and health condition of pangasius (*Pangasianodon hypophthalmus*) in a farm trial

**DOI:** 10.1016/j.aqrep.2021.100699

**Published:** 2021-07

**Authors:** Mohammad Mahfujul Haque, Neaz A. Hasan, Mahmoud M. Eltholth, Pranta Saha, Shayla Sultana Mely, Tanvir Rahman, Francis J. Murray

**Affiliations:** a Department of Aquaculture, Bangladesh Agricultural University, Mymensingh, Bangladesh; b Institute of Aquaculture, University of Stirling, Stirling FK9 4LA, United Kingdom; c Department of Hygiene and Preventive Medicine, Faculty of Veterinary Medicine, Kafrelsheikh University, Kafrelsheikh, Egypt; d Global Academy of Agriculture and Food Security, The Royal (Dick) School of Veterinary Studies and The Roslin Institute, The University of Edinburgh, Edinburgh, United Kingdom

**Keywords:** Probiotics, Pangasius, Survival, Growth performance, Hematology, Intestinal morphology

## Abstract

The effects of in-feed probiotics on growth performance, haematological parameters, gut microbial content, and morphological changes to pangasius fish were assessed. The trial had three phases, i.e., larvae to fry (20 days), fry to fingerlings (45 days), and grow-out phase from fingerlings to marketing (90 days). The stocking densities were 400 m^–3^, 200 m^–3^, and 12 m^–3^ for phases 1, 2, and 3, respectively. Phases 1 and 2 were conducted in hapas in the same pond, whereas phase 3 was performed in concrete tanks. The in-feed probiotic was administered at a rate of 0.2 g kg^–1^ of feed three times per day in phases 1 and 2 only. In phase 3, in-feed probiotics was not applied to any groups. The treated group exhibited higher growth performances (p < 0.05) than the control in all three phases of experiment. The survival % in phase 1 and 2 were found significantly (p < 0.05) higher in treatment groups. This indicates that pangasius nurserers would benefit from using probiotics as a safeguard to increase fry survival to a greater extent. Two haematological parameters including red blood cells (RBC) and white blood cells (WBC) levels were found significantly (p < 0.05) higher in treated groups in phase 2 and 3, while glucose and hemoglobin level were found significantly (p < 0.05) higher in the treated groups during phases 2 and 3, respectively. The gut microbiota content was relatively higher in the treated groups in phase 2 and 3. Histological findings indicate that the use of probiotics during the nursing phases of pangasius induced a positive change in the intestinal morphological structures. The positive impacts of probiotics on the phase 3 confirmed an immediate and long-term growth performance and health of pangasius.

## Highlights

• Growth and survival % were significantly increased in in-feed probiotics treated pangasius fry.
• Growth performances of treated groups at fingerling and grow-out phases were higher.
• Hematological parameters including hemoglobin, RBC and WBC were significantly higher in treatment groups.
• Gut microbiota content was relatively higher in probiotic treated groups at fingerling phase.
• Probiotics impact positively changed intestinal morphological structures of pangasius in treatment groups.

## 1 Introduction

Bangladesh is the 5^th^ largest aquaculture producing country that produced more than four million metric tons (MT) in 2016–2017 (DoF, 2017; FAO, 2018), and total pangasius (catfish) production reached 441,643 MT in 2017–2018, which represented 23.24 % of aquatic production (DoF, 2018). In Bangladesh, most pangasius hatcheries and some framers have nursing facilities integrated with their operation systems. The fish nursery process is an incomplete system wherein fish fries and fingerlings are raised for subsequent transfer to grow-out ponds to attain market weight. Nursery ponds are usually restocked with pangasius larvae 3–4 times per year from late March or middle April to September. The late cycle fry may be kept for a longer period to produce overwintering fingerlings for the early stocking of grow-out ponds in the subsequent year.

In Bangladesh, pangasius production cycle can be categorized into three different phases. *Phase 1– Larvae to fry*: farmers usually stock about 2 kg of larvae per 0.13 ha pond maintaining a stocking density of 15 m^−2^ where larvae are intensively fed for 10–12 days. It usually takes four weeks for the larvae to grow into fry (0.3–1.0 g). *Phase 2 – Fry to fingerlings*: the fry is transferred to one or two 0.13 ha ponds (maintaining a stocking density of 3 or 1.5 m^-2^ accordingly) depending on how many days they will be reared before sale or transfer to grow-out ponds. A high fry stocking density at this stage often results in mass mortality. This phase may take from 45 to 60 days to produce fingerlings (14–20 g). The immune system during the early stages of fingerling development remains immature and susceptible to infectious diseases (Hasan and Ahmed, 2002). *Phase 3 – Fingerlings to adult* (marketable size fish): the fingerlings are stocked for 7–8 months to achieve a marketable size (approximately one kg per fish).

The catfish juvenile production stage (phase 1) is the most vulnerable to high mortality. In this phase, surviving fish have poor health conditions and farmers often have to stock poor quality seeds, which leads to slow growth in the culture system. Potongkam and Miller (2006) reported that the catfish survival % during the first nursery phase was very low (40–50 %) compared to the second nursery phase (60–70 %). Furthermore, seeds from non-pathogen-free hatcheries or nurseries could be infected and transfer pathogens to the grow-out systems where disease can lead to high mortality rates (Faruk and Anka, 2017; Hasan et al., 2020; Hasan and Haque, 2020). This poor survival greatly affects the availability of fries that can be used to stock grow-out ponds and consequently farmers profitability (Ansa, 2014). The main reason for the high mortality is the immature immune system, which makes the early developmental stages more susceptible to infectious diseases and mortality (Chaput et al., 2020; Faruk and Anka, 2017). Farmers usually use antibiotics as a prophylactic measure to control the mortality of pangasius fries during the nursing phase, but the use of antibiotics has been discouraged after the spread of antimicrobial resistance. Therefore, farmers use other prophylactic control measures and have become increasingly reliant on PHPs, such as probiotics, prebiotics, and other immunostimulants, to promote fish health and improve innate immune system resilience (Akhter et al., 2015). Previous studies have indicated that the use of microorganisms such as *Bacillus* spp. can improve water quality, and the growth and survival of fish (Banerjee et al., 2010; Lalloo et al., 2007; Rengpipat et al., 1998). Devaraja et al. (2013) showed that beneficial bacteria, such as *Bacillus* spp., can also inhibit the growth of harmful bacteria, such as *Vibrio* spp. The use of probiotics in aquaculture has been resulted in low operational cost of farming and higher profit margin, which is about 20 % higher than the farming with no use of probiotics (El-Haroun et al., 2006). Probiotics have been used as a feed additive since the 1970s but their effects on pangasius fry are not fully understood. Probiotics were originally incorporated into the feed to increase growth and improve health by increasing resistance to disease (Fuller, 1992). According to Hai (2015), a wide range of microorganisms, including gram-negative and gram-positive bacteria, such as *Lactobacillus, Lactococcus, Leuconostoc, Enterococcus, Carnobacterium, Shewanella, Bacillus, Aeromonas, Vibrio, Enterobacter, Pseudomonas, Clostridium,* and *Saccharomyces* species, have been used as probiotics in aquaculture. Lactic acid bacteria are widely used in humans and terrestrial animals and are present in healthy fish intestines (Hagi et al., 2004; Ringø and Gatesoupe, 1998). Similarly, *Bacillus* spp. are often used in aquaculture to enhance growth performance, innate immune responses, and disease resistance (Heo et al., 2013).

Different varieties of probiotics with different compositions and modes of action are available in Bangladesh. However, their efficacy and/or optimal time and length of application are not completely understood. Therefore, the aim of this study was to assess the immediate and long-term effect of the in-feed probiotic “Sanolife PRO-F” on pangasius health and performance during the different phases of the production cycle on farm. This trial was a part of a large research project “IMAQulate project” across India, Bangladesh and Kenya funded by the Biotechnology and Biological Science Research Council (grant number BB/N005082/1) for the assessment of the effectiveness and cost benefit of prophylactic health products in aquaculture. The selection of the probiotic for this study was based on the ‘pedigree analysis tool developed under IMAQulate project’ in which the following indicators were applied; use-prevalence, declared manufacturing quality assurance certification, laboratory analysis and expert opinion on product active ingredient composition, concentration and mode of action against manufacturer and label claims. It was also based on results by other researchers (Elsabagh et al., 2018; Hostins et al., 2017; Krummenauer et al., 2020) who proved that Sanolife PRO-F improves the growth and performance of Nile tilapia and shrimp both in field as well as laboratory settings in Egypt and Brazil.

## 2 Materials and methods

### 2.1 Experimental site

This trial was divided into three phases, i.e., larvae to fry (phase 1), fry to fingerlings (phase 2), and grow-out (phase 3) where the first two phases were carried out at Rupa Hatchery and Nursery, Dhala, Mymensingh, Bangladesh over 65 days (phase 1–20 days and phase 2–45 days). This site was selected because more than 40 hatcheries are functional there and of these, 80 % were directly involved in pangasius seed production, and have nursery ponds to grow fries to fingerling. Dhala is also home to approximately 100 pangasius nurseries supplies fingerlings to aquaculture farms in larger pangasius-producing regions (Mymensingh and Comilla). The first two phases of the trial were conducted in hapas (size of each hapa: 3 × 2 × 1 m) placed in a 2024 m^2^ pond. Before these, the pond water was completely drained and all aquatic vegetation was manually removed. The pond was fenced with a synthetic net and surrounded by dykes for biosecurity and to protect against unwanted animals. Bottom racking was performed in order to remove toxic gases from the pond bottom. On day 1, diluted lime (CaCO_3_) was applied onto the pond surface at 150 kg ha^–1^. The pond was filled with water from a deep tube-well situated at the hatchery complex before setting the hapas on day 2. The grow-out phase was carried out at the Intensive Aquaculture Systems Laboratory of the Faculty of Fisheries, Bangladesh Agricultural University (BAU), Mymensingh, Bangladesh.

### 2.2 Experimental design and animals

**Fig. 1:**
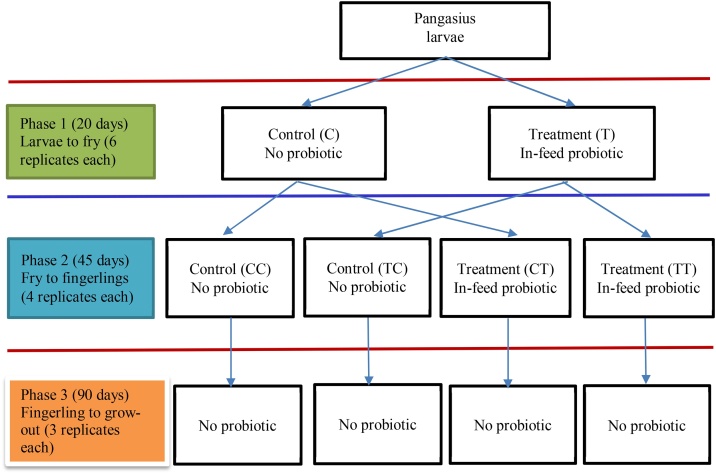
Experimental design for assessing the impact of in-feed probiotic on pangasius at different production stages.

This experiment was conducted in three consecutive phases transferring animal form one phase to another: first two phases took place in a pond and the third was performed in tanks (Fig. 1). In phase 1 (larvae to fry), 12 hapas were stocked with pangasius larvae with an average weight of 0.03 g at a stocking density of 400 m^–3^ for 20 days. Six hapas received starter feed only and the other six hapas received starter feed mixed with in-feed probiotic (Sanolife PRO-F; composed of *Bacillus subtilis, B. licheniformis*, and *B. pumilus* at a concentration of 1 × 10^10^ cfu g^–1^).

Phase 2 (fry to fingerlings) was conducted in the same nursery pond and lasted 45 days. A total of 16 fresh hapas were used and the stocking density was 200 m^–3^. Fries from the six control hapas in phase 1 were randomly reallocated into eight new hapas. Among the eight hapas, four (each treated as a replication) did not receive any Sanolife in either phase 1 or 2 (designated as control, CC). The other four hapas (each treated as replication) did not receive Sanolife in phase 1 however, received Sanolife in the phase 2 (designated as CT). In the same way, fries from the treatment hapas of phase 1 were reallocated to another eight hapas where four hapas (each treated as a replication) that were fed with Sanolife in phase 1 but not in phase 2, designated as TC. Other four received Sanolife in both phase 1 and phase 2 (designated as TT) (Fig. 1). The fish were allocated in a random manner from the phase 1 control and treatment hapas.

Phase 3 (grow-out) was conducted in 12 concrete tanks (three replications for each treatment) at the Intensive Aquaculture Systems Laboratory, BAU for 90 days. No probiotics were administered during this phase, and the stocking density was 12 m^–3^. This phase was experimented in concrete tanks to avoid any physical damage of barbels (catfish barbels are larger enough to stick in the net at grow-out stage and break while try to be free forcefully) entanglement in net and following slow painful death from fins (especially pectoral fins) damage (Price, 2016).

The stocking densities in all three phases of the trial were based on farmer practices at the field level. The source of the pangasius larvae was the Rupa Hatchery, Mymensingh, Bangladesh.

### 2.3 Feeding

Feeding and feed management in each phase were based on local aquaculture practices. For the first 2 days after stocking the hapa with larvae, each hapa received one boiled chicken egg yolk with an equal amount of wheat flour per day. The probiotic was mixed with the feed at a rate of 0.2 g kg^–1^ (Elsabagh et al., 2018) in the phase 1 and 2 treatment hapas. On day 3, a commercial nursery feed, ‘Mega Feed’, was fed at 100 % of fish body weight (BW). The feed was composed of 36.46 % crude protein, 6.35 % crude lipid, 28.66 % carbohydrates, 4.77 % crude fiber, 11.83 % ash, and 11.93 % moisture (Proximate analysis at the Laboratory of Fish Nutrition, BAU). The mixing method of probiotics in the feed and frequency of feeding fish were performed according to the manufacturer’s recommendations (Hostins et al., 2017). The required quality of probiotics was diluted in 50 mL of water and sprayed over the commercial powdered feed for homogenous mixing. Then the probiotics mixed feed was air dried for 30 min before each meal to that the bacteria get attached with feed properly. The feed was provided three times a day at 9:00, 12:00 and 16:00 h. This feeding strategy was followed during all the phases, and the feeding rate was adjusted every week after sampling and weighing the fish. In phase 3, no probiotics was mixed with the feed for all groups.

### 2.4 Growth monitoring and data collection

The length and weight of three randomly selected fish from each hapa were measured and recorded every 7 days. At the end of each phase, the weight, length, and total number of animals were used to estimate the growth and survival % in all hapas. The weight and length of three individuals from each hapa were determined using a digital balance. The growth and performance factors were calculated using the following formulae (Aanyu et al., 2018; Li et al., 2020; Zhao et al., 2021).

$$\text{L}\text{e}\text{n}\text{g}\text{t}\text{h}\;\text{g}\text{a}\text{i}\text{n}\;\left(\text{c}\text{m}\right)=(\text{M}\text{e}\text{a}\text{n}\;\text{f}\text{i}\text{n}\text{a}\text{l}\;\text{l}\text{e}\text{n}\text{g}\text{t}\text{h}-\text{M}\text{e}\text{a}\text{n}\;\text{i}\text{n}\text{i}\text{t}\text{i}\text{a}\text{l}\;\text{l}\text{e}\text{n}\text{g}\text{t}\text{h})$$

$$\text{W}\text{e}\text{i}\text{g}\text{h}\text{t}\;\text{g}\text{a}\text{i}\text{n}\;\left(\text{g}\right)=(\text{M}\text{e}\text{a}\text{n}\;\text{f}\text{i}\text{n}\text{a}\text{l}\;\text{f}\text{i}\text{s}\text{h}\;\text{w}\text{e}\text{i}\text{g}\text{h}\text{t}-\text{M}\text{e}\text{a}\text{n}\;\text{i}\text{n}\text{i}\text{t}\text{i}\text{a}\text{l}\;\text{f}\text{i}\text{s}\text{h}\;\text{w}\text{e}\text{i}\text{g}\text{h}\text{t})$$

$$\text{P}\text{e}\text{r}\text{c}\text{e}\text{n}\text{t}\;\text{w}\text{e}\text{i}\text{g}\text{h}\text{t}\;\text{g}\text{a}\text{i}\text{n}=\frac{\text{M}\text{e}\text{a}\text{n}\;\text{f}\text{i}\text{n}\text{a}\text{l}\;\text{w}\text{e}\text{i}\text{g}\text{h}\text{t}\;\left(\text{g}\right)–\;\text{M}\text{e}\text{a}\text{n}\;\text{i}\text{n}\text{i}\text{t}\text{i}\text{a}\text{l}\;\text{w}\text{e}\text{i}\text{g}\text{h}\text{t}\;(\text{g})}{\text{M}\text{e}\text{a}\text{n}\;\text{i}\text{n}\text{i}\text{t}\text{i}\text{a}\text{l}\;\text{w}\text{e}\text{i}\text{g}\text{h}\text{t}\;(\text{g})}\times 100$$

$$\text{S}\text{p}\text{e}\text{c}\text{i}\text{f}\text{i}\text{c}\;\text{g}\text{r}\text{o}\text{w}\text{t}\text{h}\;\text{r}\text{a}\text{t}\text{e}\;\left(\text{S}\text{G}\text{R})\;(\text{\%}\right)=\;\frac{\text{ln}{\text{W}}_{2}-\text{ ln}{\text{W}}_{1}}{{\text{t}}_{2}-\;{\text{t}}_{1}}\;\times 100$$

$$\text{S}\text{u}\text{r}\text{v}\text{i}\text{v}\text{a}\text{l}\;\left(\text{\%}\right)=\;\frac{\text{F}\text{i}\text{n}\text{a}\text{l}\;\text{n}\text{o}.\;\text{o}\text{f}\;\text{f}\text{i}\text{s}\text{h}}{\text{I}\text{n}\text{i}\text{t}\text{i}\text{a}\text{l}\;\text{n}\text{o}.\;\text{o}\text{f}\;\text{f}\text{i}\text{s}\text{h}}\;\times 100$$

$\text{C}\text{o}\text{n}\text{d}\text{i}\text{t}\text{i}\text{o}\text{n}\;\text{f}\text{a}\text{c}\text{t}\text{o}\text{r}\;\left(\text{K}\right)=\;\frac{\text{W}\text{e}\text{t}\;\text{b}\text{o}\text{d}\text{y}\;\text{w}\text{e}\text{i}\text{g}\text{h}\text{t}}{{\text{T}\text{o}\text{t}\text{a}\text{l}\;\text{l}\text{e}\text{n}\text{g}\text{t}\text{h}}^{3}}\;\times 100$ (Ricker, 1975)

$$\text{H}\text{e}\text{p}\text{a}\text{t}\text{o}\text{s}\text{o}\text{m}\text{a}\text{t}\text{i}\text{c}\;\text{i}\text{n}\text{d}\text{e}\text{c}\;\left(\text{H}\text{S}\text{I}\right)\;\left(\%\right)=\;\frac{\text{L}\text{i}\text{v}\text{e}\text{r}\;\text{w}\text{e}\text{i}\text{g}\text{h}\text{t}}{\text{T}\text{o}\text{t}\text{a}\text{l}\;\text{w}\text{e}\text{i}\text{g}\text{h}\text{t}}\;\times 100$$

$$\text{V}\text{i}\text{s}\text{c}\text{e}\text{r}\text{o}\text{s}\text{o}\text{m}\text{a}\text{t}\text{i}\text{c}\;\text{i}\text{n}\text{d}\text{e}\text{x}\;\left(\text{V}\text{S}\text{I}\right)\;\left(\%\right)=\;\frac{\text{V}\text{i}\text{s}\text{c}\text{e}\text{r}\text{a}\;\text{w}\text{e}\text{i}\text{g}\text{h}\text{t}}{\text{T}\text{o}\text{t}\text{a}\text{l}\;\text{w}\text{e}\text{i}\text{g}\text{h}\text{t}}\;\times 100$$

Hapa conditions were checked and cleaned every seven days. The dissolved oxygen (DO), temperature, and pH were measured directly using portable devices (Model - CE 225908 for DO, and model – CE 224469 for pH, Waltham, Massachusetts, United States) twice a day. Nitrite and total ammonia nitrogen (TAN) was measured every three days and alkalinity were measured once a week using Hach test kits (Düsseldorf, Germany).

### 2.5 Haematological studies

Haematological analyses were performed according to the protocol proposed by Horch et al. (2008). Three fish were randomly sampled from each treatment every 15 days for the haematological assessment in phase 2. The fish were caught using a small scoop net and placed on a dissection tray. Blood samples from each fish were withdrawn from the caudal vein without anesthetization using a micropipette. Whole blood was withdrawn in less than 1 min per fish to avoid stress and minimize errors in normal blood values. The parameters measured were red blood cells (RBC), white blood cells (WBC), hemoglobin, and glucose. A further smearing was performed to check the normality of the blood cells from the selected fish when compared to their blood cell abnormalities.

Blood samples for estimating the WBC count were collected in sterilized Eppendorf tubes containing anticoagulant (ethylenediaminetetraacetic acid (EDTA)) and WBC dilution fluid (glacial acetic acid, gentian violet-1% w/v, and distilled water). Samples for RBC counts were collected in Eppendorf tubes containing EDTA and RBC dilution fluid (mercuric chloride, sodium sulfate, sodium chloride, and distilled water). All blood samples were transferred to the laboratory in an icebox. WBCs and RBCs were counted within two hours of sampling. The samples were placed on a Neubauer hemocytometer (EMS Sedgewick-Rafter Counting Cell, Brass, 1.0 mL, 63509−01) and observed using a light microscope (Primo Star HAL/LED microscope) attached to a camera (Zesis, Axiocam ERc 5 s) at 4× and 10× magnifications. WBC and RBC counts were then estimated using the following equations:

$$\text{W}\text{B}\text{C}\;(\times 10^{3}/{\text{m}\text{m}}^{3})=\;\frac{\text{T}\text{o}\text{t}\text{a}\text{l}\;\text{n}\text{u}\text{m}\text{b}\text{e}\text{r}\;\text{o}\text{f}\;\text{c}\text{e}\text{l}\text{l}\;\text{i}\text{n}\;1\;\text{l}\text{a}\text{r}\text{g}\text{e}\;\text{s}\text{q}\text{u}\text{a}\text{r}\text{e}\text{s}\;\times \text{d}\text{i}\text{l}\text{l}\text{u}\text{t}\text{i}\text{o}\text{n}\;\text{f}\text{a}\text{c}\text{t}\text{o}\text{r}\;\times \text{c}\text{o}\text{u}\text{n}\text{t}\text{i}\text{n}\text{g}\;\text{f}\text{a}\text{c}\text{t}\text{o}\text{r}\text{s}}{\text{V}\text{o}\text{l}\text{u}\text{m}\text{e}\;\text{f}\text{a}\text{c}\text{t}\text{o}\text{r}\;(0.1)}$$

$$\text{R}\text{B}\text{C}\;\left(\times 10^{6}/{\text{m}\text{m}}^{3}\right)=\;\frac{\text{T}\text{o}\text{t}\text{a}\text{l}\;\text{n}\text{u}\text{m}\text{b}\text{e}\text{r}\;\text{o}\text{f}\;\text{c}\text{e}\text{l}\text{l}\;\text{i}\text{n}\;5\;\text{l}\text{a}\text{r}\text{g}\text{e}\;\text{s}\text{q}\text{u}\text{a}\text{r}\text{e}\text{s}\;\times \text{d}\text{i}\text{l}\text{u}\text{t}\text{i}\text{o}\text{n}\;\text{f}\text{a}\text{c}\text{t}\text{o}\text{r}\;\times \text{d}\text{e}\text{p}\text{t}\text{h}\;\text{f}\text{a}\text{c}\text{t}\text{o}\text{r}\text{s}}{\text{N}\text{u}\text{m}\text{b}\text{e}\text{r}\;\text{o}\text{f}\;\text{s}\text{m}\text{a}\text{l}\text{l}\;\text{s}\text{q}\text{u}\text{a}\text{r}\text{e}\;\text{c}\text{o}\text{u}\text{n}\text{t}\text{e}\text{d}}\;\times 16$$

Samples for hemoglobin (Hb) estimation were collected in Eppendorf tubes containing Hb fluid (hydrochloric N/10). Hb was measured using a Sahli hemometer in accordance with the method proposed by Drabkin (1946) and the Hb content was expressed as g%.

Blood samples were processed and used to determine plasma glucose according to Bartoňková et al. (2016) using a Fish Glucose Transporter 2 ELISA kit and the absorbance was measured using a plate reader (Easymate® GHb). Blood smears were prepared according to Fenech et al. (2003). The slides were stained with Gimsa for approximately 12 min, thoroughly washed in distilled water, and dried at room temperature. The slides were observed under a light microscope (Micros Austria/MCX100 microscope) with a computer connected camera (AmScope, CMOS C-mount) at 40× magnification.

### 2.6 Enumeration of gut microbiota from experimental fish

Fish samples (three per treatment) were collected, packed in ice boxes and transported to the laboratory within 3 h to isolate the gut microbiota content. After surface sterilization of the fish, the entire gut was carefully removed and homogenized in sterile physiological saline (0.85 %). The resultant aliquot was serially diluted, plated on nutrient agar (NA) and de Man, Rogosa, and Sharpe (MRS) agar (Hi-media, India) prepared according to the manufacturer’s instructions, and incubated for 24 h at 37 °C to recover the total heterotrophic bacteria (THB) in the gut samples. The gut sample bacterial populations were expressed as numbers of colony forming units mL^–1^ (cfu mL^–1^).

### 2.7 Histological observation

The first histopathology sample was collected after 8 days of stocking in phase 1. The fry ranged from 7.3 to 12 mg in weight and 8–11 mm in length. Three fish were randomly collected from each replicate, and one fish was randomly selected from the three. In phase 1, whole fish was sampled. In phase 3, a second sample was taken and the fry range was 16.7–74.5 g in weight and 13.5–19.5 cm in length. Samples were collected from the gill, intestine, kidney, spleen, and liver. Intestinal samples were collected from three different parts of the intestine. These were the foregut (1 cm; begins after the esophageal opening and continues until the start of the hepatic loop), the midgut (coiled part of the intestine), and the hindgut (1 cm; starts after the coiled part and continues up to 2 cm before the anus) according to the method proposed by Tan et al. (2016) and Coz-Rakovac et al. (2008).

All samples were washed in 1.2 % saline solution, fixed in 4% paraformaldehyde solution for 48 h, and then washed in 70 % ethanol solution. The same 70 % ethanol solution was used to store the samples until processing. The final paraffin production, image collection, and sample measurement processes were performed according to Torrecillas et al. (2007); Xu et al. (2016), and Ye et al. (2016), respectively.

### 2.8 Statistical analysis

One-way analysis of variance (ANOVA) was used to determine significant differences (p < 0.05) among treatments for pangasius growth performance, survival, and bacterial abundance in water. The Bonferroni test was applied when significant differences were detected. The histological data from the first phase was statistically compared using a two-sided, independent sample *t*-test with a 0.05 (5%) level of significance. Histological data from the third phase were subjected to one-way ANOVA to determine significant (p < 0.05) variations among the various parameters in the experimental groups. IBM SPSS Statistics for Windows, Version 23.0 (Armonk, NY, USA) was used for all analyses.

## 3 Results

### 3.1 Growth and survival

#### 3.1.1 Phase 1

The final weight, weight gain, average daily growth, and SGR were non-significantly higher in the treatment group than those in the control group (Table 1). However, final length, length gain, condition factor and survival % were significantly higher (p < 0.05) in the treated group than in the control group.

**Table 1:** Growth performance of pangasius (*Pangasianodon hypophthalmus*) treated with in-feed probiotic versus no treatment during the phase 1 (20 days), phase 2 (45 days) and phase 3 (90 days).

Growth parameters	*Phase 1*			*Phase 2*					*Phase 3*				
	C	T	*p* value	TT	TC	CT	CC	*p* value	TT	TC	CT	CC	*p* value
Final weight, g	0.30 (0.44)	0.53 (0.72)	0.244	14.31 (10.23)	11.58 (12.33)	11.05 (12.58)	10.78 (12.09)	0.889	38.01 (1.68)	42.48 (4.63)	35.89 (2.36)	35.16 (6.59)	0.229
Final length, cm	2.14 (1.24)	3.16 (1.42)	0.028	10.25 (2.94)	8.44 (4.35)	8.02 (5.03)	7.47 (4.72)	0.461	14.67 (0.24)	14.74 (0.91)	14.21 (0.37)	14.33 (0.86)	0.723
Weight gain, g	0.27 (0.40)	0.50 (0.70)	0.244	13.78 (10.23)	11.05 (12.33)	10.75 (12.58)	10.48 (12.09)	0.904	22.15 (3.09)	27.27 (4.41)	21.11 (4.50)	22.03 (6.91)	0.456
Length gain, cm	1.70 (1.20)	2.70 (1.40)	0.028	7.09 (2.94)	5.28 (4.35)	5.88 (5.03)	5.33 (4.72)	0.716	4.67 (0.95)	3.41 (0.59)	4.24 (2.12)	5.83 (0.58)	0.191
Condition factor	2.26 (0.95)	1.24 (0.59)	0.001	1.30 (0.40)	2.77 (2.57)	3.90 (3.94)	4.30 (4.01)	0.102	1.20 (0.01)	1.31 (0.13)	1.25 (0.02)	1.18 (0.09)	0.242
Average daily growth, g	0.01 (0.02)	0.03 (0.04)	0.244	0.31 (0.23)	0.25 (0.27)	0.24 (0.28)	0.23 (0.27)	0.904	0.25 (0.03)	0.30 (0.05)	0.23 (0.05)	0.24 (0.08)	0.456
Specific growth rate, %	21.21 (5.87)	24.59 (6.21)	0.102	6.94 (1.29)	6.22 (1.55)	7.13 (1.93)	7.04 (1.98)	0.563	1.08 (0.06)	1.42 (0.11)	1.30 (0.08)	1.29 (0.21)	0.061
Survival, %	30.05 (1.28)	40.27 (1.71)	0.001	58.58 (1.25)	52.25 (1.18)	44.83 (2.81)	28.73 (1.30)	0.001	97.00 (5.19)	83.00 (8.00)	80.33 (4.62)	83.00 (8.00)	0.056

Values are means of three replications and SDs are shown in parentheses. Different superscript letters within rows indicate significant differences (p < 0.05).

#### 3.1.2 Phase 2

Except survival % there were no significant (p > 0.05) differences between the treatments and control. However, in most cases, all parameters related to growth performance, except condition factor and SGR, were found relatively higher in the TT group followed by TC, CT, and CC (control). The difference between the groups (TT and TC) that had received Sanolife in phase 1 was larger than the difference between the groups (CT and CC) that had not received Sanolife in phase 1. This indicated that the use of probiotics in the early phase of fingerling rearing may have a longer-term impact on the growth of pangasius fry. As with phase 1, there were significant (p < 0.05) differences among the groups and the survival % was significantly higher in the TT group than those in the other groups (Table 1).

#### 3.1.3 Phase 3

Similar to phase 2, there were no significant (p > 0.05) differences of growth performance among the treatment and control groups in phase 3. However, final weight and length in the TC and TT groups were higher than in the other groups where TC and CT showed the highest CF and SGR (Table 1). Interestingly, the greatest length gain was attained in the CC group and the lowest in TC, while the largest weight gain was observed in TC and lowest in CT. The survival of pangasius in TT was found to be higher than the other groups.

### 3.2 Haematological results

There were significant (p < 0.05) differences among the treatments for glucose level, RBC, and WBC. However, there were no significant differences among the fingerling hemoglobin levels in phase 2 (Table 2). The higher RBC levels in the TT and TC groups indicated that in-feed probiotics can enhance fish immunity, which would significantly improve fingerling survival. With the exception of glucose level, significant differences were observed between the treatment and control groups for the same haematological parameters in phase 3. Overall, TT was the highest performer among the treatment groups, while the lowest was the control group (Table 2).

**Table 2:** Haematological parameters of pangasius (*Pangasianodon hypophthalmus*) treated with in-feed probiotic versus no treatment for samples collected at 45^th^ and 90^th^ day of culture.

Experimental phase	Parameters	TT	TC	CT	CC	*P* value
**Phase 2**	Glucose level	119.13 (18.51)^a,^*	152.00 (17.77)^b^	106.67 (12.50)	121.00 (11.79)^a^	0.036
	Hemoglobin level	4.67 (0.24)	5.27 (0.23)	4.60 (1.20)	4.53 (1.45)	0.739
	RBC** (10^6^ mm^−3^)	1.49 (0.07)^a^	1.01 (0.05)^ab^	0.82 (0.08)^b^	0.59 (0.11)^a^	0.010
	WBC*** (10^5^ mm^−3^)	1.64 (0.11)^a^	1.33 (0.13)^b^	1.02 (0.08)^a^	0.75 (0.09)^a^	0.041
**Phase 3**	Glucose level	121.17 (39.36)	110.33 (27.81)	98.83 (15.80)	83.5 (14.85)	0.579
	Hemoglobin level	4.05 (0.21)^a^	3.9 (0.14)^a^	3.55 (0.07)	3.1 (0.14)^b^	0.011
	RBC (10^6^ mm^−3^)	3.97 (0.13)^a^	2.95 (0.11)^ab^	2.01 (0.11)^a^	1.31 (0.15)^a^	0.022
	WBC (10^5^ mm^−3^)	1.89 (0.12)^a^	1.49 (0.07)^a^	1.18 (0.09)^b^	0.79 (0.09)^c^	0.003

* Values are the means of three replications and SDs are shown in parentheses. Different superscript letters within rows indicate significant differences (p < 0.05).

** RBC – Red blood cell.

*** WBC – White blood cell.

### 3.3 Fingerling gut microbiota

The NA gut microbiota count was relatively higher in TC than in the other treatment and control groups during phase 2. In phase 3, TT was the highest reading counter group and the lowest was CT. For MRS agar, the highest gut microbiota count was also the TC group during phase 2, whereas the CT and CC treatment counts were below average. The highest MRS agar gut microbial reading in phase 3 was observed in the TT group and the lowest reading was recorded by the CC group (Table 3).

**Table 3:** Average total plate count (cfu/mL) of gut microbiota at phase 2 and phase 3.

Experimental phase	Treatments							
	TT		TC		CT		CC	
	NA*	MRS**	NA	MRS	NA	MRS	NA	MRS
Phase 2	9.00×$10^{5}$	2.00×$10^{2}$	6.10×$10^{6}$	2.50×$10^{2}$	1.70×$10^{5}$	<10	5.50×$10^{4}$	<10
Phase 3	6.35×$10^{6}$	6.40×$10^{3}$	3.20×$10^{5}$	1.45×$10^{3}$	2.05×$10^{5}$	6.50×$10^{2}$	2.85×$10^{5}$	1.50×$10^{2}$

* NA – Nutrient agar.

** MRS – de Man, Rogosa, and Sharpe agar.

### 3.4 Histological findings

**Fig. 2:**
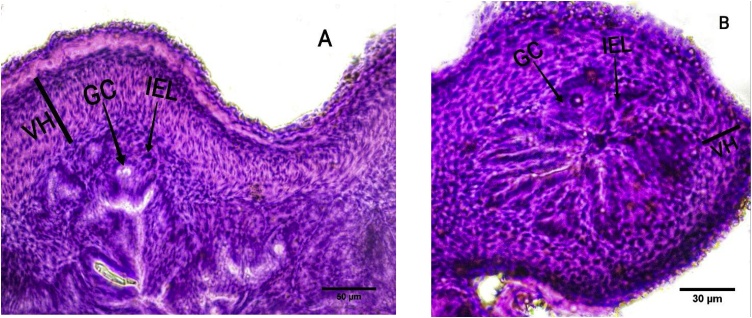
Microscopic image of the intestinal morphology of first stage fish. Distal intestinal part with the indication of villus height (VH), goblet cell (GC) and intra epithelial lymphocytes (IEL) for the fish fed with treatment diet. Scale bar =50 μm (A). Distal part of intestine with the indication of VH, GC and IEL for fish fed with control diet. Scale bar =30 μm (B). Specific counting was done randomly selected 1 × 1 μm^2^ section from the image for specific count of each treatment and control group fish in ImajeJ.

**Fig. 3:**
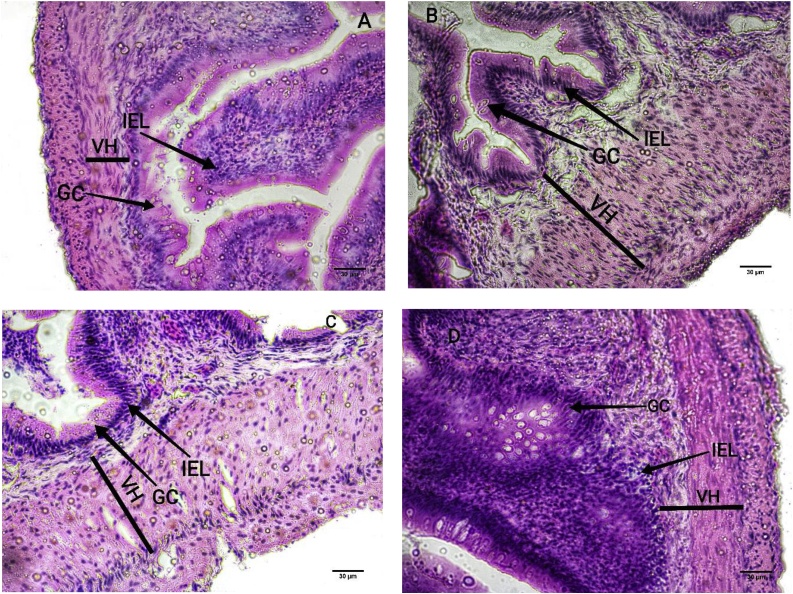
Microscopic image of the intestinal morphology of third stage fish. Middle intestinal part with the indication of villus height (VH), goblet cell (GC) and intra epithelial lymphocytes (IEL) for the fish of CC group. Scale bar =30 μm (A). Middle part of intestine with the indication of VH, GC and IEL for CT group fish. Scale bar =30 μm (B). Middle intestinal part with the indication of villus height VH, GC and IEL for the fish of TC group. Scale bar =30 μm (C). Middle part of intestine with the indication of VH, GC and IEL for TT group fish. Scale bar =30 μm (D). Specific counting was done randomly selected 1 × 1 μm^2^ section from the image for specific count of each treatment and the following control group fish in ImajeJ.

The intestinal villous height, cellular distribution of mucus-secreting cells (goblet cells) and intra-epithelial lymphocytes (IEL) showed a marked difference in regional distribution and localization along the intestinal length between the treatment and control group fingerlings in phase 1 (Fig. 2). Fishes from the treatment and control groups also showed marked regional distribution differences and localizations for intestinal villous height, cellular distribution of mucus-secreting cells (goblet cells), and IEL in phase 3 (Fig. 3).

#### 3.4.1 Intestinal villus height

The villous heights in all three parts of the intestine in phase 1 for the probiotic treated fry were significantly higher (p < 0.05) than in the control group. The results were the same in phase 3. The absorptive area of the intestine was significantly higher in TC (p < 0.05) than in the other groups (Table 4).

**Table 4:** Histological findings in different intestinal parts of pangasius (*Pangasianodon hypophthalmus*) treated with or without probiotic in different phases of growth.

Intestinal villus height (μm)								
	Phase 1		*p value*	Phase 3				*p value*
*Part of the intestine*	T	C		TT	TC	CT	CC	
Proximal part	498.18	381.60	< 0.001	469.20	802.28	570.80	417.88	< 0.001
Middle part	478.06	333.50	< 0.001	527.96	848.33	596.72	463.09	< 0.001
Distal part	426.40	293.65	< 0.001	459	779.85	565.63	419.53	< 0.001

Goblet cells								
	Phase 1		*p value*	Phase 3				*p value*
*Part of the intestine*	T	C		TT	TC	CT	CC	
Proximal part	18	14	0.123	22	32	18	14	0.001
Middle part	16	10	0.102	19	25	15	13	0.01
Distal part	12	9	0.218	13	23	12	10	0.011

Intra epithelial lymphocyte								
	Phase 1		*p value*	Phase 3				*p value*
*Part of the intestine*	T	C		TT	TC	CT	CC	
Proximal part	39	29	0.029	38	36	19	15	<0.001
Middle part	34	24	0.031	31	29	17	13	0.002
Distal part	27	20	0.033	25	21	12	9	0.024

#### 3.4.2 Goblet cells

The number of goblet cells in the intestine was comparatively, but not significantly higher, in the treatment group than in the control group during phase 1. During phase 3, the goblet cell numbers in the intestine were significantly higher in the TC group (p < 0.05) than in the other groups (Table 4).

#### 3.4.3 Intra epithelial lymphocytes

The probiotic-fed fish had a significantly (p < 0.05) higher number of IEL than the control group in the intestine during phase 1. This indicated that there had been a qualitative change in the in-feed probiotic group compared to the control group. In phase 3, the number of intestinal IEL was higher in the TT group than in the other groups (p < 0.05) (Table 4).

### 3.5 Water quality parameters

During the trial phases, the average water temperatures of the pond and tanks were 27.48 ± 0.397 °C and 19.06 ± 0.285 °C, respectively. The minimum water temperature was 18.2 °C and the maximum water temperature was 28.5 °C. The mean values for DO in the water were 5.24 ± 0.177 and 4.76 ± 0.175, respectively, and the mean pH values were 7.32 ± 0.159 and 7.64 ± 0.130 in the ponds and tanks, respectively.

## 4 Discussion

In aquaculture, exposure to stressful conditions and problems related to diseases often occur and these lead to high mortality and serious economic loss. Over recent decades, probiotics have increasingly been used to mitigate stress and pathogens via different mechanisms, such as improving digestion by supplying exoenzymes and the establishment of beneficial microflora in the digestive tract. Various studies have reported on the use of probiotics in finfish and shellfish aquaculture as alternatives to antibiotics (Chauhan and Singh, 2019; Hoseinifar et al., 2018), to enhance growth (Cruz et al., 2012; Ibrahem, 2013), to increase feed efficiency (Ahmed et al., 2015, 2014; Nargesi et al., 2020) and improve fish production safety (Dawood et al., 2019; Mohapatra et al., 2013). However, the application of probiotics at the nursing stage to assess their immediate impact on fish survival (catfish fries are vulnerable at the nursery stage) and their long-term effect on health and growth performance remains undocumented.

Our results showed that the final length, length gain, and condition factor values were significantly higher in the treatment group than in the control group during phase 1, but they were not significantly different in the other two phases. The condition factor is a very much interconnected with the weight–length ratio (Giosa et al., 2014). It represents the physio-biological condition of fish and fluctuates depending on feeding conditions, disease occurrence, and physiological factors (Le Cren, 1951). The high condition factor in the phase 1 treatment group showed that the application of a probiotic populated by *Bacillus* species ensured good health conditions and isometric growth during the pangasius nursing phase. Opiyo et al. (2019a) also found that the use of *Bacillus* spp. improved the condition factor of *Oreochromis niloticus* fry. However, Opiyo et al. (2019b) concluded that *Bacillus* spp. did not improve the condition factor of *O. niloticus* fry. Furthermore, Azarin et al. (2014) suggested that the high condition factor in the control group compared to the *Bacillus* treated *Rutilus frisii kutum* fry may have been because the treated group were negatively affected by other physicochemical factors associated with the rearing systems. The results from this study showed that applying a *Bacillus* based probiotic significantly improved *P. hypophthalmus* survival during both the nursing and fingerling phases. Survival at the grow-out phase was higher in the TT group than in the other groups. Sahoo et al. (2015) conducted a three-phase experiment to observe the growth and survival of sun catfish. Mortality was highest during the first phase of the experiment, i.e. the larval stage. These studies suggest that catfish nurseries are the most vulnerable stakeholders in the fish value chain because of the high mortality during the phases that they are associated with (Loc et al., 2010). The administration of *Bacillus* as a probiotic may reduce mortality through fast larval growth, which would be similar to the report on *Centropomus undecimalis* by Tarnecki et al. (2019). Sayes et al. (2018) strongly recommended the use of probiotic bacteria to improve fish larval survival. For example, Dennis and Uchenna (2016) and Hauville et al. (2016) showed that adding mixed *Bacillus* species to the first feeds improved African catfish and snook catfish survival, respectively. Therefore, adding a mixed *Bacillus* based probiotic can be a cost-effective solution for small-scale catfish farmers in Bangladesh at the nursery level. It is possible to treat a large number of fish at the larval stage with minimal amounts of probiotics and at low cost. This means that hatchery and nursery operators can reduce the likelihood of fish diseases and mental disturbance in subsequent phases.

The application of a *Bacillus* spp. probiotic significantly improved the RBC and WBC counts of the treatment groups in phases 2 and 3. The RBCs and WBCs are the two most important blood cells for fish oxygen circulation in the respiratory system, blood flow regulation (Jensen, 2009), and innate and adaptive immunity (Uribe et al., 2011). Mixed *Bacillus* strain probiotics have been shown to improve the haematological profiles of *O. niloticus* (Elsabagh et al., 2018; Opiyo et al., 2019a) and rainbow trout (Capkin and Altinok, 2009). Glucose levels in blood samples from the TC group in phase 2 and hemoglobin in the TT group were significantly different from those in the other groups. Like RBCs, hemoglobin is also involved in oxygen dispersion throughout the body (Riggs, 1970) and it helps fish to adapt to continuous changes in metabolic requirements and the environment (Powers, 1980). Akanmu (2018) suggested that probiotics are the best chemotherapeutants for improving haematological parameters because they enhance fish immune and stress responses. Telli et al. (2014) and Hassaan et al. (2014) also found that *Bacillus* probiotics improved the haematological indices in *O. niloticus*. Therefore, *Bacillus* strains can be considered as potential probiotics for the enhancement of fish welfare from nursing to the grow-out stages in aquaculture farming.

Microbiological results indicate that *P. hypophthalmus* gut microbiota also improved in the treatment groups when the fish were fed a mixed *Bacillus* based probiotic during phase 2. The gut microbiota also improved in the phase 3 in treatment groups where the fish had previously been treated with the probiotic throughout phase 2, even though no probiotic was supplied to any treatment group in phase 3. This means that the effect of probiotic *Bacillus* species may last for some time when fish are treated with probiotics at the early stages of growth. These probiotics may promote fish defense mechanisms, which ultimately improve fish survival over the production cycle. Previous studies (Asaduzzaman et al., 2018; Ramos et al., 2013; Siriyappagouder et al., 2018) reported that probiotics had short term effects on *Tor tambroides*, *Oncorhynchus mykiss*, and zebrafish. However, Yeh et al. (2020) reported that probiotics had long-term effects in oyster aquaculture. Therefore, probiotics containing *Bacillus* species can be considered as effective probiotics because they improve the intestinal environment of fish through biological conditioning and control agents.

The mixed *Bacillus* probiotic did not markedly affect goblet cells during the initial nursing phase. However, a long-term goblet cell count effect was observed during the grow-out phase when the fish were treated in phase 2. The primary function of the fish intestine is digestion and the absorption of food (Wilson and Castro, 2010), and it is secondarily considered as a first-line barrier against infection (Firdaus-Nawi et al., 2013; Secombes and Ellis, 2012). The surface area of the intestine becomes larger when the villus height increases, which boosts intestinal performance. Increasing the goblet cell counts in different fish organs promotes the secretion of mucus and some immunological substances (Concha et al., 2003; Fernandes et al., 2004; Lindenstrøm et al., 2003; Tsutsui et al., 2005), which directly or indirectly protect the fish from pathogens (Cain et al., 1996). Furthermore, an increase in intraepithelial lymphocytes with developmental, morphological, and functional features (Scapigliati et al., 2018) enhances phagocytosis through the secretion of antibodies (Firdaus-Nawi et al., 2013). The histological findings of this study further prove that applying probiotics containing mixed *Bacillus* strains at the catfish larval stage can extend the profit margins of farmers by boosting the survival % through improvements to fish defense mechanisms. The results from this study confirm those reported by Abdel-Aziz et al. (2020) who showed that the selection of effective microorganisms as probiotics for *O. niloticus* improved their survival % and exponentially increased intraepithelial lymphocyte and goblet cell counts.

## 5 Conclusion

The pangasius catfish is an important farmed species and contributes the most to aquaculture production in Bangladesh. However, larval rearing of catfish at the nursery level is affected by high mortality, which has led to poor hatchery and nursery production efficiencies. The results from this study show that mixed *Bacillus* based probiotics reduce pangasius fry mortality and that hatchery and nursery owners can use them as a natural alternative to antibiotics during the first phase to improve fish growth and production.

## Ethical considerations

This study was conducted in accordance with the Ethical Standard of Research Committee of Bangladesh Agricultural Research System (BAURES), Bangladesh Agricultural University, Mymensingh.

## Data availability statement

The data that support the findings of this study are available from the corresponding author upon request.

## Author Statement

**Mohammad Mahfujul Haque:** Conceptualization of the study, drafting manuscript, data analysis, results interpretation, editing and organizing the final version of the manuscript.

**Neaz A. Hasan:** Implementation of the study, drafting manuscript, histological analysis, data processing and analysis, results interpretation, editing and organizing the final version of the manuscript.

**Mahmoud M. Eltholth:** Conceptualization of the study, data analysis, results interpretation, editing and organizing the final version of the manuscript.

**Pranta Saha:** Implementation of the study, data recording, data processing, literature review and initial drafting manuscript.

**Shayla Sultana Mely:** Implementation of the study, hematological analysis, data recording, literature review and drafting manuscript.

**Tanvir Rahman:** Hematological analysis, microbial study, and initial draft of the manuscript.

**Francis J. Murray:** Conceptualization of the study, experimental design, editing and organizing the final version of the manuscript.

## Declaration of Competing Interest

The authors declare no conflict of interest.
